# Regulatory effect of Phikud Navakot extract on *HMG-CoA reductase* and *LDL-R*: potential and alternate agents for lowering blood cholesterol

**DOI:** 10.1186/s12906-018-2327-1

**Published:** 2018-09-24

**Authors:** Napatara Tirawanchai, Sudarat Supapornhemin, Anchaleekorn Somkasetrin, Bhoom Suktitipat, Sumate Ampawong

**Affiliations:** 1 Department of Biochemistry, Faculty of Medicine Siriraj Hospital, Mahidol University, 2 Bangkok Noi Road, Bangkok Noi, Bangkok, 10700 Thailand; 2 Department of Tropical Pathology, Faculty of Tropical Medicine, Mahidol University, 420/6 Ratchawithi Road, Ratchathewi, Bangkok, 10400 Thailand

**Keywords:** Cholesterol, HMG-CoA reductase, Low-density lipoprotein receptor, Phikud Navakot, Ya-Hom Navakot

## Abstract

**Background:**

For decades, various cardiovascular symptoms have been relieved by the use of Ya-Hom Navakot, which is a formulation comprising 54 herbal medicines. The Thailand Ministry of Public Health listed Ya-Hom Navakot’s nine active principle and nomenclative herbal ingredients and termed them ‘Phikud Navakot’ (PN). Several reports have confirmed that PN has cardiovascular benefits similar to Ya-Hom Navakot. However, whether PN facilitates lipid-lowering activity remains unclear.

**Methods:**

The present study investigated an in vitro model for examining the gene expression levels of 3-hydroxyl-3-methylglutaryl-CoA reductase (*HMGCR*) and low-density lipoprotein receptor (*LDL-R*) in HepG2 cells using qRT-PCR. The ethanol and water extractions of Ya-Hom Navakot, PN and Ya-Hom Navakot without PN were compared.

**Results:**

One mg/ml of both NYEF and NYWF were found to significantly lower cholesterol by either the up-regulation of *LDL-R* or down-regulation of *HMGCR* compared with negative controls and 1 mg/ml simvastatin (*p* < 0.05). PNEF also up-regulated *LDL-R* gene expression, even more than NYEF (*p* < 0.05). In addition, the ethanol and water extracts of PN significantly down-regulated *HMGCR* gene expression compared with those of Ya-Hom Navakot without PN (*p* < 0.05).

**Conclusion:**

The use of Ya-Hom Navakot or PN may provide an alternative treatment to lower cholesterol through *HMGCR* gene inhibition and *LDL-R* gene enhancement.

## Background

In Thailand, traditional medicines are used in primary healthcare for treating illnesses and diseases because of their effectiveness and minimal side effects. Thai traditional medicines and Thai herbal formulae are promoted by the Thailand Ministry of Public Health for use as alternative medicine in the treatment of health problems. Thai herbal medicines are recorded in the List of Herbal Medicinal Products (LHMP) A.D. 2012, which contains numerous polyherbal formulations. All polyherbal formulations are considered to provide maximal therapeutic efficacy with less toxicity [1].

Ya-Hom Navakot is a well-known Thai polyherbal formulation that originated as a product of Thai wisdom. It comprises 54 herbal medicines and is frequently used in primary healthcare and Thai traditional household. Ya-Hom Navakot improves blood circulation and reduces dizziness, nausea and vomiting [2]. However, when the Ministry of Public Health included Ya-Hom Navakot in LHMP A.D. 2012, the 54 herbal medicines were reduced to nine principle herbal ingredients and termed as ‘Phikud Navakot’ (PN), while the rest were excluded. All the nine herbs are mixed in equal proportions and include the following: Kot Soa (*Angelica dahurica*), Kot Chiang (*A. sinensis*), Kot Kradook (*Saussurea costus*), Kot Khamao (*Atractylodes lancea*), Kot Huabua (*Ligusticum chuanxiong*), Kot Kanprao (*Picrorhiza kurrooa*), Kot Jatamansi (*Nardostachys jatamansi*), Kot Chulalumpa (*Artemisia pallens*) and Kot Pungpla (*Terminalia chebula*).

Even without the 45 excluded herbal plants of Ya-Hom Navakot, it is still claimed that PN improves the functioning of the cardiovascular system. Moreover, PN is reported to be effective in improving blood circulation [3]. The hydro-ethanolic extract of PN significantly attenuates carbachol-induced vasorelaxation in endothelium-intact rat aorta, partly through its antagonistic effect on the muscarinic receptor [2]. Moreover, the hydro-ethanolic extract of PN possesses antioxidant properties; it finds reactive oxygen species (ROS) and reactive nitrogen species (RNS) in human endothelial ECV304 cells more effectively than the water-extract of PN [4]. The antioxidant properties of PN also preserve the integrity and osmotic ability of red blood cells throughout the induced oxidative stress [5]. Furthermore, it is considered safe because no treatment-related mortality events have been observed among acute and sub-chronic toxicity studies in PN-fed rats [6].

Based on the documented effects of PN, it is also possible that Ya-Hom Navakot has a lipid-lowering activity. In the present study, the expression of the genes encoding 3-hydroxyl-3-methylglutaryl-CoA reductase (*HMGCR*) and low-density lipoprotein receptor (*LDL-R*), both of which are crucial enhancing factors for cholesterol biosynthesis, were examined in HepG2 cell lines for demonstrating the hypocholesterolaemic activities of PN and Ya-Hom Navakot. In vitro cultures and quantitative reverse transcription-polymerase chain reaction (qRT-PCR) were used. The results provide a better understanding of the effect of PN on blood lipid homeostasis and propose it as a candidate for anti-lipidaemic therapy or adjunct treatment for patients with hypercholesterolaemia.

## Methods

### Reagents

Dulbecco’s modified Eagle’s medium (DMEM), minimal essential medium (MEM), foetal bovine serum (FBS), penicillin–streptomycin, glutamine, non-essential amino acids and sodium pyruvate were purchased from GIBCO Laboratories (Grand Island, NY, USA). Dimethyl sulfoxide (DMSO) was obtained from Prolabo (Paris, France). Simvastatin (Zocor®) was obtained from Berlin Pharmaceutical Co. Ltd. (Bangkok, Thailand). GENEzol™ reagents, which were used for RNA extraction, were obtained from Geneaid (New Taipei, Taiwan). Reagents for first-strand cDNA synthesis were available at Thermo Fischer Scientific (Waltham, MA, USA). FastStart Essential DNA Green Master Kit, which was used in qRT-PCR, was obtained from Roche (Mannheim, Germany). MTT [3-(4, 5 di-methylthiazol-2-yl)-2, 5-diphenyltetrazolium bromide] was purchased from Sigma-Aldrich (St. Louis, MO, USA). All other chemical reagents were of analytical grade and highest quality.

### Plant materials and preparation of PN extracts

The roots of *A. dahurica* (Fisch.) Benth, et Hook. f. (Apiaceae), *A. sinensis* (Oliv.) Diels (Apiaceae) and *S. costus* (Falc.) Lipsch. (Asteraceae), rhizomes of *A. lancea* (Thunb.) DC. (Asteraceae), *L. chuanxiong* Hort. (Apiaceae) and *P. kurrooa* Royle ex Benth. (Scrophulariaceae), roots and rhizomes of *N. jatamansi* (D. Don) DC. (Valerianaceae), aerial parts of *A. pallens* Walls ex DC. (Asteraceae) and galls of *T. chebula* Retz. (Combretaceae) were purchased in October 2009 from traditional drugstores in Bangkok, Thailand. Dr. Sanya Hokputsa, who is affiliated with the Research and Development Institute, Government Pharmaceutical Organisation, examined all the specimens. Voucher specimens (NVK10–52) have been deposited at the Phytochemical Research Group, Research and Development Institute, Government Pharmaceutical Organisation, Thailand. All herbal materials were examined according to the quality control parameters of Thai Herbal Pharmacopoeia and compared with authentic specimens, which were generously provided by Associate Professor Dr. Noppamas Soonthornchareonnon, Faculty of Pharmacy, Mahidol University, Thailand.

PN extracts were prepared as previously described by Nalintara [4] using ethanol reflux as demonstrated in the previous report [7]. Briefly, each dried plant material was powdered and equally mixed before sieving through a No. 40 mesh. The powdered herbs (1 kg each) were extracted with 2 × 5 L of either 50% ethanol or water (PNEF and PNWF, respectively) under reflux for 3 h. Extracts were then spray-dried or freeze-dried. Stock solutions of the extracted PN were prepared by dissolving 1 g of the extract in 5 ml of 100% DMSO to a final concentration of 200 mg/ml. Aliquots of the stock solutions were prepared and stored at − 20 °C until use.

### Ethanolic and water extracts of Ya-Hom Navakot polyherbal formulation

In October 2009, all the 54 herbs included in the Ya-Hom Navakot polyherbal formulation were purchased from traditional drugstores in Bangkok, Thailand, examined by Dr. Sanya Hokputsa, dried and mixed. The herbal mixtures were extracted using 50% ethanol or water (NYEF and NYWF, respectively) as previously described. For control, plant materials excluding PN were prepared and extracted using 50% ethanol (NBEF) or water (NBWF).

### In vitro culture of HepG2 cells

HepG2 cell lines purchased from American Type Culture Collection (ATCC, HB 8065) were cultured in DMEM containing MEM supplemented with 10% FBS, penicillin (100 units/ml), streptomycin (100 μg/ml), 2.0 mM glutamine, 0.1 mM non-essential amino acids and 1.0 mM sodium pyruvate. The cells were cultured at 37 °C under 5% CO_2_ and 98% relative humidity until they reached 80% confluence.

### MTT assay

The viability of HepG2 cells was assessed using the MTT assay [8, 9]. Briefly, HepG2 cells were plated at a density of 1 × 10^4^ cells/well in 100-μl DMEM complete medium in a 96-multiwell plate and cultured at 37 °C under 5% CO_2_ and 98% relative humidity for 24 h. DMEM complete medium was aspirated before treatment with a test medium containing the DMEM complete medium and standard concentrations (0.01, 0.05, 0.1, 0.5 and 1 mg/ml) of PNEF, PNWF, NYEF, NYWF, NBEF, or NBWF for 24 h. The experimental medium was aspirated, and MTT solution was added to each well at a concentration of 0.25 mg/ml before incubation for another 2 h at 37 °C. After the addition of 100-μl DMSO/well, the absorbance of purple formazan at 570 nm was determined. Simvastatin was used as the positive control. Cytotoxicity for all herbal extracts and their respective concentrations was calculated using the percentage of living cells in relation to the percentage of cells treated with only 0.5% DMSO (negative control).Cytotoxicity of all herbal extracts was calculated as the percentage of cell viability using the following equation:

$$ \frac{\%\mathrm{cell}\ \mathrm{viability}=\left({\mathrm{OD}}_{570}\mathrm{of}\ \mathrm{the}\ \mathrm{test}\ \mathrm{condition}-{\mathrm{OD}}_{570}\ \mathrm{of}\ \mathrm{the}\ \mathrm{blank}\right)\ x\ 100}{\left({\mathrm{OD}}_{570}\ \mathrm{of}\ \mathrm{the}\ \mathrm{standard}\ \mathrm{control}-{\mathrm{OD}}_{570}\ \mathrm{of}\ \mathrm{the}\ \mathrm{blank}\right)} $$

### RNA preparation and qRT-PCR

For assessing the effect of PNEF, PNWF, NYEF, NYWF, NBEF and NBWF on the expressions of *HMGCR* and *LDL-R*, HepG2 cells were seeded at 1 × 10^6^ cells/ml and cultured in DMEM complete medium for 24 h before treatment with 1 mg/ml of herbal extracts. The cells were also treated with 0.5% DMSO and 1 mg/ml of simvastatin as negative and positive controls, respectively. After 24 h of treatment, total RNA was extracted using GENEzol™ reagents following the manufacturer’s protocol. The concentration of the extracted RNA was measured using a spectrophotometer (NanoPhotometer™, Implen GmBH, Munich, Germany). One microgram of total RNA was subjected to reverse transcription to obtain cDNA using the RevertAid First-Strand cDNA Synthesis Kit (Thermo Fischer Scientific) according to the manufacturer’s instructions. qRT-PCR was performed using the FastStart Essential DNA Green Master Kit (Roche, Mannheim, Germany) according to the manufacturer’s instructions. qRT-PCR included a heat inactivation step at 95 °C for 10 min, followed by 40 cycles of denaturation at 95 °C for 30 s, annealing at 60 °C for 45 s and extension at 72 °C for 1 min using a Stratagene quantitative PCR Mx3005 (Stratagen, USA). Oligonucleotide primers designed using OLIGO 7 primer analysis software were ordered from Sigma-Aldrich (Singapore). For the amplification of *HMGCR*, forward and reverse primers used were 5′-TACCATGTCAGGGGTACGTC-3′ and 5′-CAAGCCTAGAGACATAAT-3′’, respectively (amplicon size = 247 bp). In addition, forward and reverse primers for amplifying *LDL-R* were 5′-TGCAGTGGGCGACAGATGCG-3′ and 5′-GGTTGACACGGCCCCCACAG-3′, respectively (amplicon size = 179 bp). The expression of each specific gene was normalised to that of the housekeeping gene GAPDH using the forward and reverse primers 5′-CAGCCTCAAGATCATCAGCA-3′ and 5′-CATGAGTCCTTCCACGATAC-3′ (amplicon size = 100 bp).

### Statistical analysis

All the results were expressed as the mean ± standard deviation (SD) from at least three independent experiments. Prism software package version 6.01 and GraphPad Software Inc. (CA, USA) were used for both statistical analysis and graph plotting. Unpaired Student’s *t-tests* or one-way analysis of variance with Tukey’s post-hoc test were used for determining statistical significance. A *p*-value of < 0.05 with 95% confidence interval was considered statistically significant.

## Results

### Cytotoxicity of all herbal extracts in HepG2 cells

**Fig. 1:**
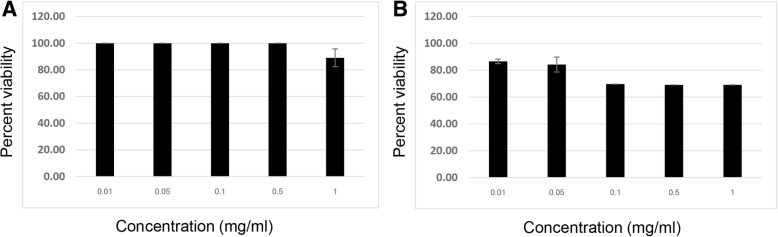
Effect of PNEF (**a**) and PNWF (**b**) on the viability of HepG2 cells assessed by the MTT assay: Cells were treated with standard concentrations (0.01, 0.05, 0.1, 0.5 and 1 mg/ml) of PNEF and PNWF for 24 h. The results are expressed as the mean ± SD from three independent experiments. DMSO (0.5%) was used as the negative control

**Fig. 2:**
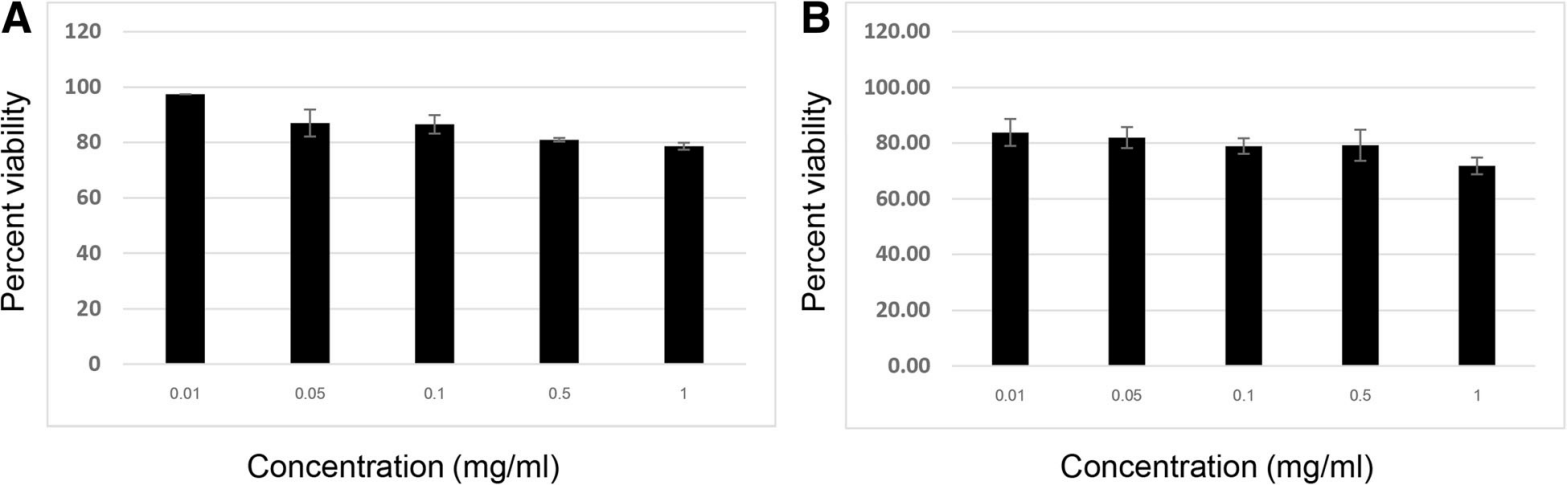
Effect of NYEF (**a**) and NYWF (**b**) on the viability of HepG2 cells assessed by the MTT assay: Cells were treated with standard concentrations (0.01, 0.05, 0.1, 0.5 and 1 mg/ml) of NYEF and NYWF for 24 h. The results are expressed as the mean ± SD from three independent experiments. DMSO (0.5%) was used as the negative control

**Fig. 3:**
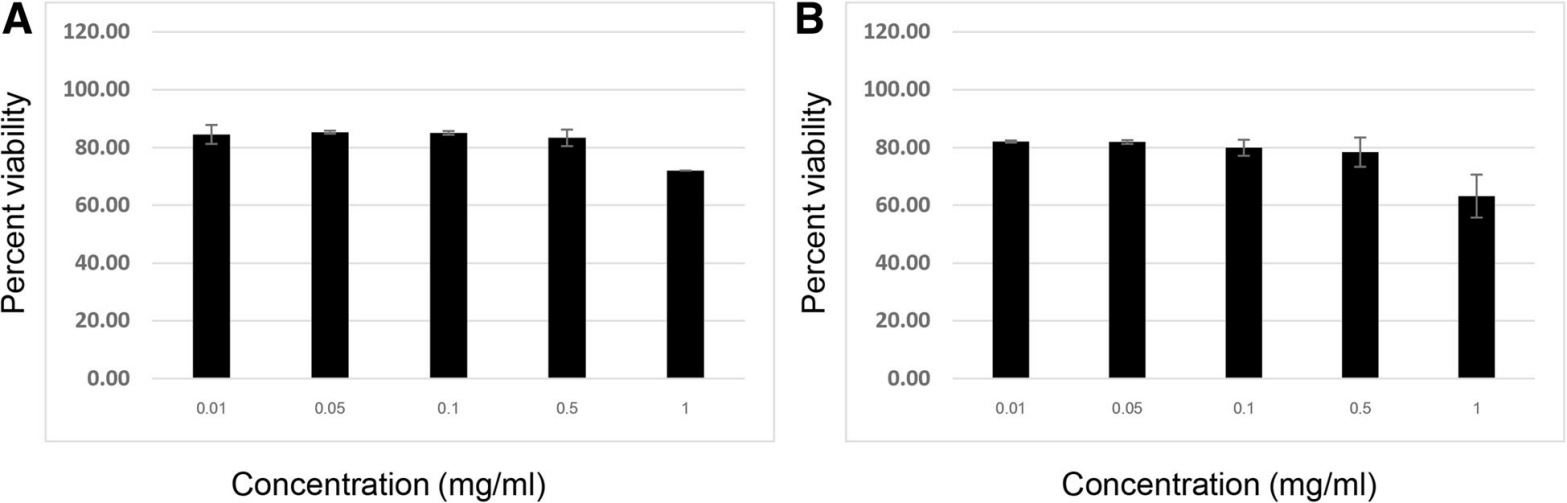
Effect of NBEF (**a**) and NBWF (**b**) on the viability of HepG2 cells assessed by the MTT assay: Cells were treated with standard concentrations (0.01, 0.05, 0.1, 0.5 and 1 mg/ml) of NBEF and NBWF for 24 h. The results are expressed as the mean ± SD from three independent experiments. DMSO (0.5%) was used as the negative control

**Fig. 4:**
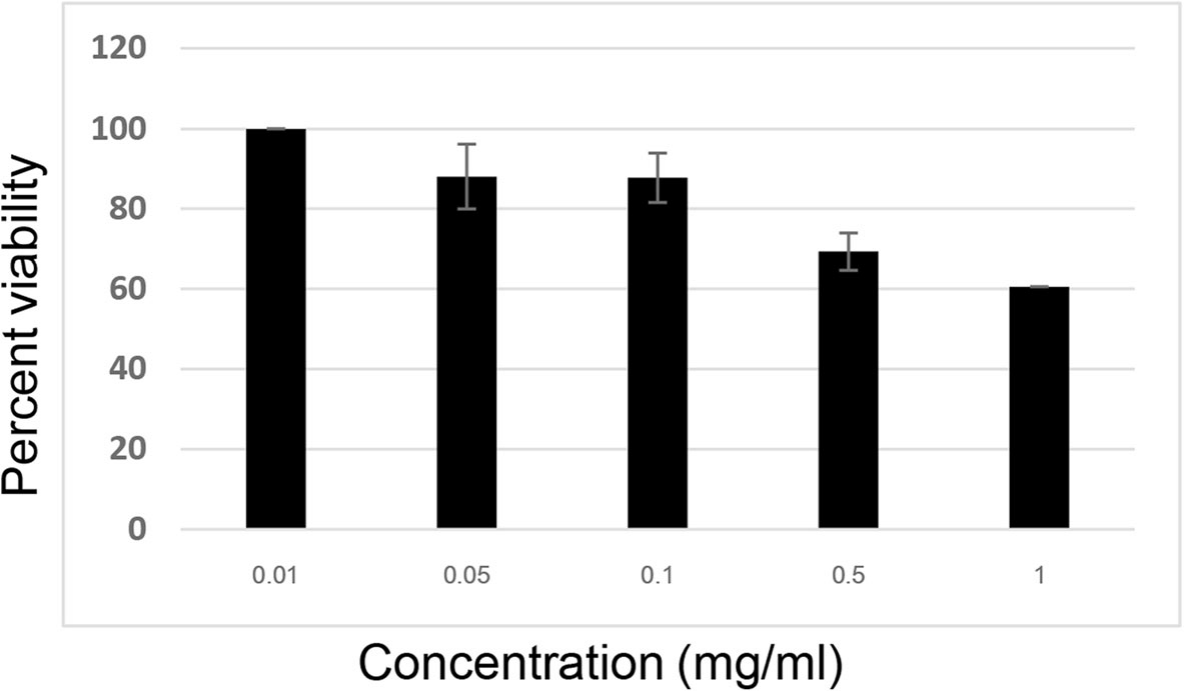
Effect of simvastatin on the viability of HepG2 cells assessed by the MTT assay: Cells were treated with standard concentrations (0.01, 0.05, 0.1, 0.5 and 1 mg/ml) of simvastatin for 24 h. The results are expressed as the mean ± SD from three independent experiments. DMSO (0.5%) was used as the negative control

The cytotoxicity of PNEF, PNWF, NYEF, NYWF, NBEF, NBWF and simvastatin in HepG2 cells was assessed using the MTT assay. Results were generated from independent experiments performed in triplicate (Figs. 1, 2, 3 and 4). Inhibitory concentration at 50% (IC_50_) for each assay was calculated using the MTT assay. No cytotoxicity was noted for any herbal extract examined in HepG2 cells (IC_50_ ≥ 1 mg/ml).

### Effect of all herbal extracts on *LDL-R* and *HMGCR* gene expression

**Fig. 5:**
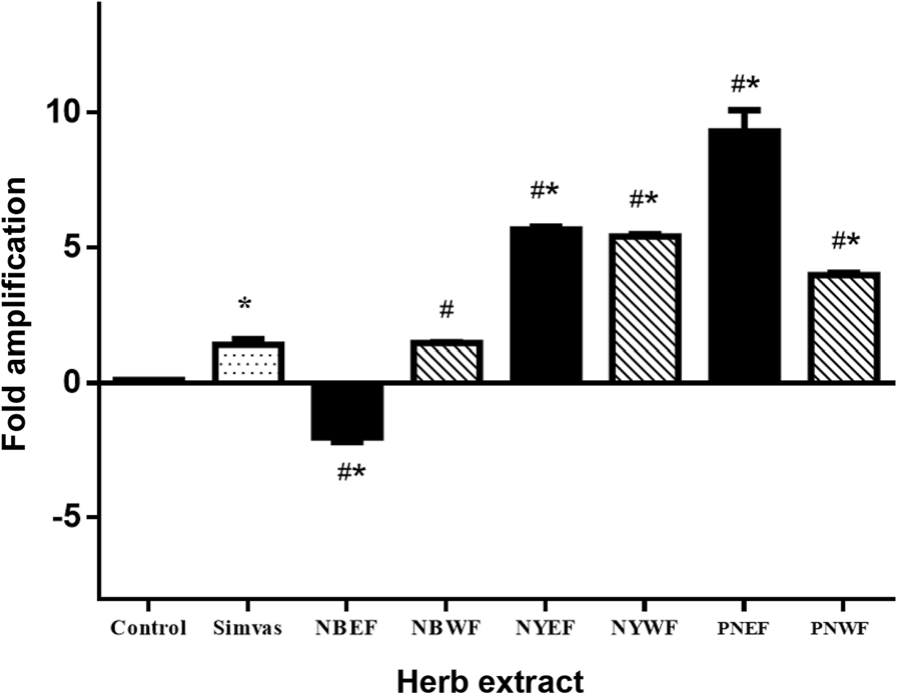
Effect of all extractions on *LDL-R* gene expression: NBEF, NBWF, NYEF, NYWF, PNEF and PNWF (1 mg/ml each) were individually added to the cells. The expression of *LDL-R* in HepG2 cells was compared with that in the presence of 1 mg/ml simvastatin and 0.5% DMSO as positive and negative controls, respectively. Bars represent the mean ± SD of independent triplicate experiments. ^#^*p* < 0.05 compared with the negative control ^*^p < 0.05 compared with simvastatin

**Fig. 6:**
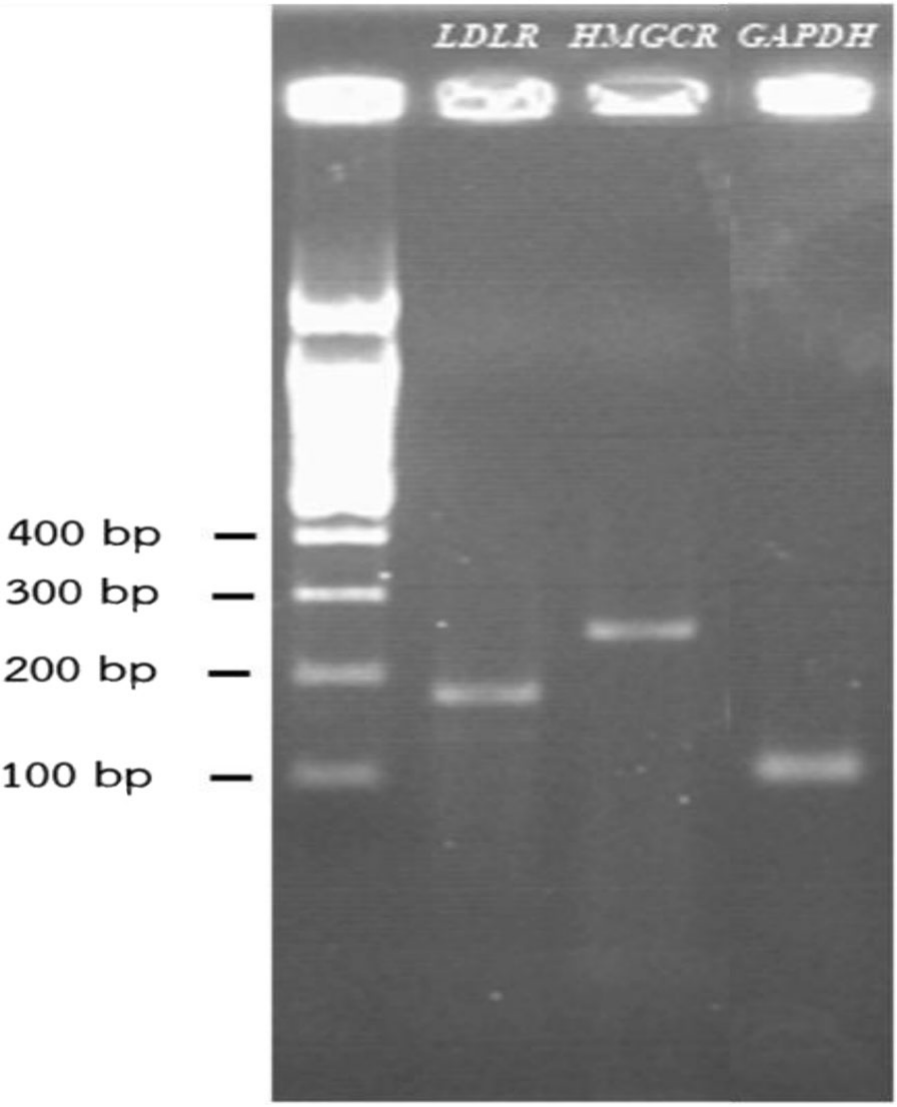
Agarose gel electrophoresis (1%) of the qRT-PCR-amplified products of *LDL-R*, *HMGCR* and *GAPDH*. Lane 1: 100-bp DNA ladder, lane 2: *LDL-R* (179 bp), lane 3: *HMGCR* (247 bp) and lane 4: *GAPDH* (100 bp)

The effect of PNEF, PNWF, NYEF, NYWF, NBEF and NBWF on *LDL-R* transcripts was assessed using qRT-PCR (Fig. 5). Furthermore, qRT-PCR-amplified DNA was detected using agarose gel electrophoresis (Fig. 6). We found that 1 mg/ml of PNEF, PNWF, NYEF and NYWF were effective and significantly enhanced the synthesis of *LDL-R* compared with the negative control DMSO and simvastatin (PNEF = 9.31 ± 0.77, NYEF = 5.68 ± 0.11, PNWF = 3.98 ± 0.08, NYWF = 5.41 ± 0.09 and simvastatin = 1.40 ± 0.21). *LDL-R* expression was up-regulated the most when using PNEF. The effects of NBEF and NBWF on *LDL-R* were intermittent, indicating that PN plays a major role in either regulating or modulating cholesterol-lowering effects and the associated gene expression.

**Fig. 7:**
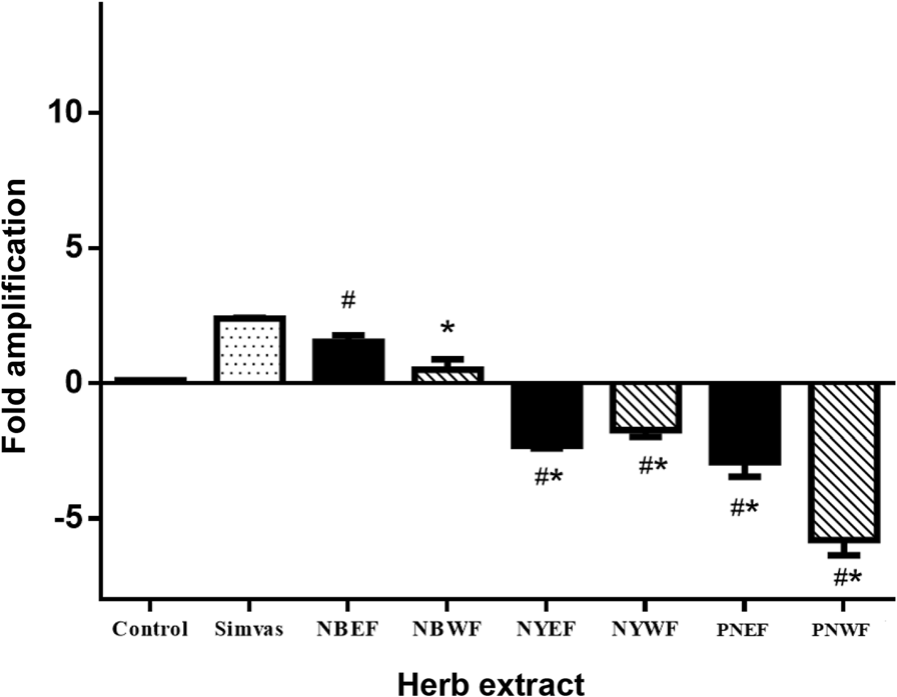
Effect of all extractions on *HMGCR*: NBEF, NBWF, NYEF, NYWF, PNEF and PNWF (1 mg/ml each) were individually added to the cells. The expression of *HMGCR* in HepG2 cells was compared with that in the presence of 1 mg/ml simvastatin and 0.5% DMSO as positive and negative controls, respectively. Bars represent the mean ± SD of independent triplicate experiments. ^#^*p* < 0.05 compared with NBEF ^*^p < 0.05 compared with NBWF

Similar to *LDL-R*, the effects of 1 mg/ml of PN (PNEF and PNWF), Ya-Hom Navakot (NYEF and NYWF) and Ya-Hom Navakot without PN (NBEF and NBWF) on *HMGCR* transcripts were determined using qRT-PCR (Fig. 7). The qRT-PCR-amplified DNA was also detected using agarose gel electrophoresis (Fig. 6). PNEF, PNWF, NYEF and NYWF significantly inhibited the expression of *HMGCR* compared with the negative control (PNEF = − 2.91 ± 0.53, PNWF = − 5.8 ± 0.57, NYEF = − 2.3 ± 0.1 and NYWF = − 1.74 ± 0.24) and down-regulated the synthesis of *HMGCR* compared with simvastatin (2.41 ± 0.03).

On the contrary, 1 mg/ml extracts of NBEF and NBWF significantly up-regulated the expression of *HMGCR* compared with the DMSO negative control (NBEF = 1.54 ± 0.25 and NBWF = 0.51 ± 0.38). However, the *HMGCR* gene expression level was lower than that observed using simvastatin (2.41 ± 0.03). Unlike *LDL-R*, PNWF exhibited a higher potential for reducing *HMGCR* gene expression than its ethanolic extract and other groups. Therefore, PN may play an important role in the inhibition of *HMGCR* gene expression.

## Discussion

The incidence of cardiovascular diseases (CVDs) has been increasing worldwide. In 2015, it was estimated that there were 422.7 million cases of CVD and 17.92 million CVD-related deaths [10]. Hypercholesterolaemia is a major risk factor for CVD progression and is rapidly becoming more prevalent in developing countries. Interestingly, in 2016, ischaemic heart disease, which is a CVD, was the primary cause of mortality in Thailand. The incidence of hypercholesterolaemia is increasing in Thailand owing to changes in the lifestyle and behaviour to adapt to the Western culture [11]. Approximately 14% and 17% of Thai men and women have hypercholesterolaemia, respectively [12]. Cholesterol biosynthesis primarily occurs in the liver as a result of the HMGCR enzyme, which is a regulatory enzyme in the mevalonate pathway [13]. This enzyme is considered a therapeutic target for lowering blood cholesterol.

Low-density lipoprotein-cholesterol (LDL-C), which is an important atherogenic lipoprotein, is metabolised in the liver by LDL-R. High levels of LDL-C in the blood can increase the risk of atherosclerosis and CVD [14, 15]. High levels of LDL-C are present in 29.6% of Thai adults [16]. As such, LDL-C has largely replaced total cholesterol as a risk marker and primary treatment target for hyperlipidaemia [17]. A reduction in LDL-C levels can lead to a reduced risk of CVD development [18].

Simvastatin, which is a widely used lipid-lowering drug, increases the mRNA synthesis of *HMGCR* and *LDL-R* [19]. It is a synthetic derivative of the fermentation product of *Aspergillus terreus* [20] and blocks cholesterol synthesis by the competitive inhibition of the HMGCR enzyme, the rate-limiting step of cholesterol biosynthesis in the human body. Simvastatin is a powerful lipid-lowering drug, primarily used for treating dyslipidaemia and preventing atherosclerosis-related complications in high-risk individuals. However, statin therapies are associated with numerous adverse effects [21–28]. Hence, alternative treatments to alleviate hyperlipidaemic conditions and substitute statins are warranted.

Several herbal extracts have been shown to affect the synthesis of HMGCR and LDL-R genes. A phenol-enriched extract of *Moringa oleifera* leaf significantly increased the expression of both *HMGCR* and *LDL-R* in HepG2 cells [29]. GINST, a hydrolysed ginseng extract, also decreased the expression of *HMGCR* via AMPKα activation in HepG2 cells [30]. In addition, hydrolic extract of lemongrass [*Cymbopogon citratus* (DC) Stapf.] suppressed the expression of sterol regulatory element binding protein-1c and *HMGCR* in rats [31]. Furthermore, low concentrations of anthocyanin (200 mg/L) extracted from Thai black sticky rice significantly enhanced the expression of *LDL-R* in HepG2 cells [32]. In this study, both PNEF and NYEF demonstrated a significant down-regulation of *HMGCR* mRNA and up-regulation of *LDL-R* mRNA compared with simvastatin. However, NBEF and NBWF effects did not differ from those of simvastatin.

In this study, different methods of extraction led to a variety of effects on target genes, depending on the composition and proportion of the extracted ingredients. In agreement with other studies, active ingredients extracted using different solvents resulted in different effects. For example, many phenolic compounds are extracted using chloroform, n-butanol and ethyl acetate rather than ethanol, ethanol/water or water [33, 34]. In this study, PNEF up-regulated *LDL-R* gene expression the most, whereas PNWF down-regulated *HMGCR* gene expression (Figs. 5 and 7). This demonstrated the board spectrum of activity when using different extraction methods. In addition, PNEF scavenged ROS and RNS better than PNWF [4].

Thus, it is suggested that PN contains herbs with the following two mechanisms involving cholesterol metabolism: (i) up-regulation of *LDL-R* resulting in the increased uptake of LDL-C and (ii) down-regulation of *HMGCR* resulting in the suppression of cholesterol biosynthesis. Although PNWF had the highest ability to decrease *HMGCR* gene expression compared with other groups, NYEF and PNEF still exhibited better effects on cholesterol metabolism than NBEF, NBWF, simvastatin and DMSO negative control. Indeed, ethanol extraction should be considered as the appropriate method for herbal extraction because it is safe, quick, consumes little energy and preserves heat-labile components. However, the mechanisms underlying the increased expression of *LDL-R* or decreased expression of *HMGCR* remain unknown.

Recently, traditional Thai medicines and herbal formulae have been promoted by the Ministry of Public Health for use as alternative medicines in the treatment of health problems [3]. Ya-Hom Navakot is one of the Thai herbal formulae listed in the Herbal Medicinal Products A.D. 2006 announced by the Ministry of Public Health. Based on traditional knowledge, it has a therapeutic effectiveness against circulatory disorders. It effectively improves blood circulation in the body through its cholesterol-lowering effect. However, the mechanisms involved in this hypocholesterolaemic effect of Ya-Hom Navakot or PN are yet to be clarified. To the best of our knowledge, this study is the first report describing the cholesterol-lowering effect of both Ya-Hom Navakot and its PN formulation and their association with *HMGCR* down-regulation and *LDL-R* up-regulation.

## Conclusion

Ya-Hom Navakot and its PN formulation create a cholesterol-lowering effect, primarily by inhibiting *HMGCR* and enhancing *LDL-R* gene expressions. The effect of these herbal extracts is greater than that of the standard cholesterol treatment using statins. Ethanol-extracted PN up-regulated *LDL-R* gene expression best, while Ya-Hom Navakot without PN down-regulated both *HMGCR* and *LDL-R* gene expressions.

## Abbreviations

DMEM: Dulbecco’s modified Eagle’s medium
LDL-C: Low-density lipoprotein-cholesterol
*A. dahurica*: *Angelica dahurica*
*A. lancea*: *Atractylodes lancea*
*A. pallens*: *Artemisia pallens*
*A. sinensis*: *Angelica sinensis*
CA: California
CVDs: Cardiovascular diseases
DMSO: Dimethyl sulfoxide
FBS: Foetal bovine serum
HMGCR: 3-hydroxyl-3-methylglutaryl-CoA reductase
IC_50_: Inhibitory concentration at 50%
*L. chuanxiong*: *Ligusticum chuanxiong*
LDL-R: Low-density lipoprotein receptor
LHMP: List of Herbal Medicinal Products
MA: Massachusetts
MEM: Minimal Essential Medium
MO: Missouri
MTT: 3-(4, 5 di-methylthiazol-2-yl)-2, 5-diphenyltetrazolium bromide
*N. jatamansi*: *Nardostachys jatamansi*
NBEF: Ya-Hom Navakot without PN ethanolic extraction
NBWF: Ya-Hom Navakot without PN water extraction
NY: New York
NYEF: Ya-Hom Navakot ethanolic extraction
NYWF: Ya-Hom Navakot water extraction
*P. kurrooa*: *Picrorhiza kurrooa*
PN: Phikud Navakot
PNEF: Phikud Navakot ethanolic extraction
PNWF: Phikud Navakot water extraction
qRT-PCR: Quantitative reverse transcription-polymerase chain reaction
RNS: Reactive nitrogen species
ROS: Reactive oxygen species
*S. costus*: *Saussurea costus*
*T. chebula*: *Terminalia chebula*
USA: United States of America
